# Midurethral sling incision: indications and outcomes

**DOI:** 10.1007/s00192-012-1895-8

**Published:** 2012-08-09

**Authors:** Volker Viereck, Oliver Rautenberg, Jacek Kociszewski, Susanne Grothey, JoEllen Welter, Jakob Eberhard

**Affiliations:** 1 Department of Gynecology and Obstetrics, Cantonal Hospital Frauenfeld, Postfach, 8501 Frauenfeld, Switzerland; 2 Department of Gynecology and Obstetrics, Georg August University, Goettingen, Germany; 3 Department of Gynecology and Obstetrics, Evangelisches Krankenhaus Hagen-Haspe, Hagen, Germany

**Keywords:** Dystopic sling position, Obstructive and non-obstructive long-term sling complications, Pelvic floor ultrasound, Sling incision, Tension-free vaginal tape (TVT), Voiding disorders

## Abstract

**Introduction and hypothesis:**

Controversy continues over the effectiveness of sling incision, which is the most common operative approach to treating complications following suburethral sling insertion. This retrospective analysis assessed the indications for sling incision and patient outcomes regarding resolution of complications and stress urinary incontinence.

**Methods:**

A review was conducted of the medical records of women who underwent sling incision between 2003 and 2010. Data such as surgical indications, ultrasound findings and medical outcomes were extracted from 198 records, and descriptive and inferential statistical methods of analysis were used.

**Results:**

In the 198 patients eligible for study inclusion, the primary reasons for sling incision were overactive bladder (68 %), voiding dysfunction (61 %), and recurrent urinary tract infections (53 %). Additional complications included dyspareunia (18 %), chronic pelvic pain (17 %), and sling exposure (15 %). Sling incision led to immediate postoperative cure of voiding dysfunction in 97 % of patients. Cure rates for overactive bladder and dyspareunia were 60 % and 94 % respectively. Chronic pelvic pain was resolved in 82 % of cases and all cases of sling exposure were cured. Eighty-five (61 %) of the 140 patients who were continent before sling incision developed recurrent stress urinary incontinence (SUI) postoperatively.

**Conclusions:**

These findings indicate that sling incision can be highly successful in improving voiding dysfunction and dyspareunia, and moderately successful in curing overactive bladder and chronic pain. However, SUI may recur in more than 60 % of the patients undergoing sling incision. Consequently, patients being considered for a sling incision procedure should be informed about this possible complication.

## Introduction

Suburethral sling insertion, tension-free vaginal tape (TVT), and transobturator tension-free vaginal tape (TVT-O) are highly successful, minimally invasive alternatives to open surgery for treating female stress urinary incontinence (SUI) [1]. Although complications are rare, intraoperative bladder perforation and postoperative obstructions causing voiding dysfunction and overactive bladder (OAB) are the most commonly identified complications [2]. As reported in the literature, voiding dysfunction occurs in 5–12 % of cases, and exacerbation or de novo overactive bladder occurs in 3–25 % of cases [3]. Patients with these complications report poor urinary flow, staccato voiding, urge symptoms, and recurrent urinary tract infections (UTI). Less common are non-obstructive complications such as sling exposure, dyspareunia, and chronic pelvic pain in the area of the sling. The inherent risk of exerting too much tension on the sling as it is being inserted poses a challenge for physicians as they attempt to balance curing the original problem of incontinence with unintentionally causing postoperative complications from dystopic or too tightly placed slings.

The time frame in which sling-related complications develop can vary considerably. Some patients experience symptoms immediately following the procedure; however, others may develop complications years later. Many women with complaints related to sling procedures can be treated conservatively. However, if the problem persists, sling incision offers the best chance of cure for most patients. Rates of sling incision procedures reported in the literature range from 1 to 20 % [4, 5].

To better understand the effectiveness of sling incision in resolving postoperative complications and treating SUI, we performed a retrospective analysis of patients who underwent such a procedure. Our primary aims were to identify indications for this type of surgical intervention and the impact of the surgery on resolving complications and the SUI recurrence rate. In addition, we analyzed the possible effects of sling position on the occurrence of complications by using the pelvic floor ultrasound method described in our previous publications [6, 7], as well as other diagnostic tools.

## Materials and methods

This retrospective analysis includes data obtained from medical records of women who underwent sling incision at a tertiary urogynecological center between January 2003 and June 2010. A detailed description of the primary complaint or indication for surgical intervention (i.e., obstructive and/or irritative bladder symptoms, sling exposure, chronic pelvic pain, dyspareunia) was obtained from each patient’s medical records. In all cases, symptoms reported by patients were verified by urogynecological examinations including pelvic floor ultrasound and urodynamic testing, which involved urethrocystoscopy.

**Fig. 1:**
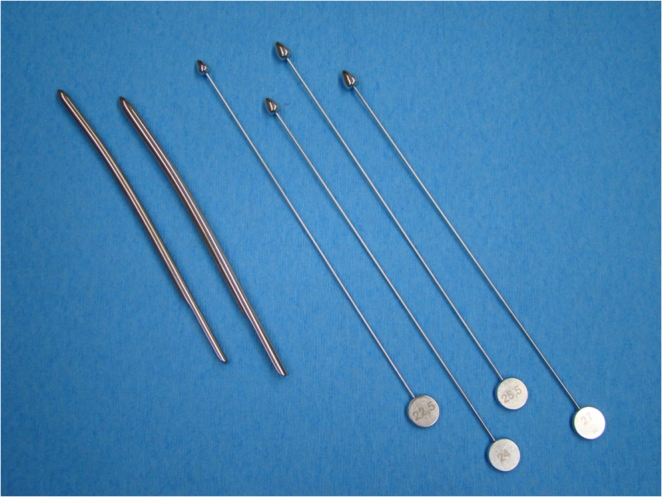
Simple diagnostic tools for supplementary diagnostic evaluation in patients with obstructive complications. Hegar dilators (*left*; optimal are Hegar Nos. with diameters of 5 to 7 mm) are used to demonstrate a stop cock phenomenon as a sign of an excessively tense sling. A bougie à boule (*right*; optimal are Charrière Nos. with circumferences of 21.0 to 24.0 mm) is used to identify a sling too close to the urethra or a penetrating sling

**Fig. 2:**
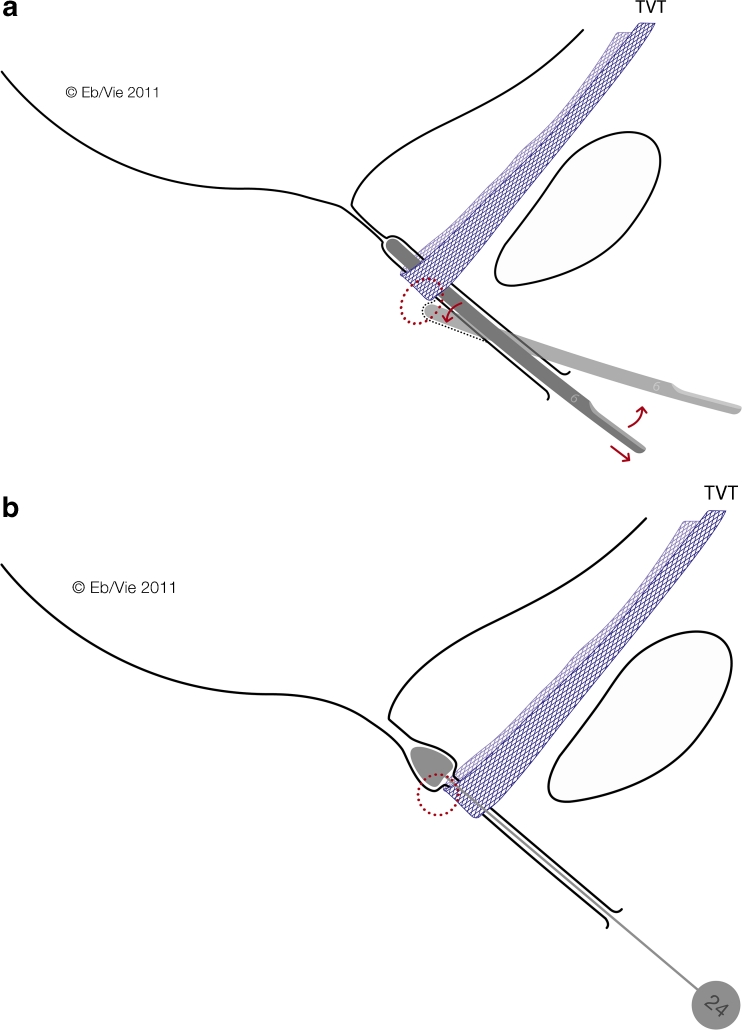
Mechanisms of simple tools for the diagnosis of obstructive complications. Hegar dilators Nos. 5 to 7 and bougies à boule Charrière 21 to 24. **a** Rotation of the Hegar dilator during retraction through the urethra will show a snap phenomenon if the sling is too tight, i.e., not tension-free in patients presenting with signs of obstructive sling complications. **b** Retraction of the bougie à boule exerting slight pressure on the posterior urethral wall will elicit severe pain at the proximal edge of the sling in patients with a sling penetrating the urethral lumen or lying very close to the lumen

Depending on patients’ complaints, supplementary diagnostic tests such as urethral calibration with a Charrière 21 to 24 bougie à boule or urethral probing/rotation using a No. 5 to 7 Hegar urethral dilator (Figs. 1, 2) were conducted. These diagnostic tools are used to determine if the urethral lumen is being compressed/obstructed by the sling and to identify the location of the compression. Residual urine volume was determined either by single catheterization during urodynamic or cystoscopic evaluation or by ultrasound. Voiding dysfunction was defined as abnormally slow and/or incomplete micturition based on subjective complaints plus objective assessment of a persistent postmicturition residual volume of greater than 100 ml.

**Fig. 3:**
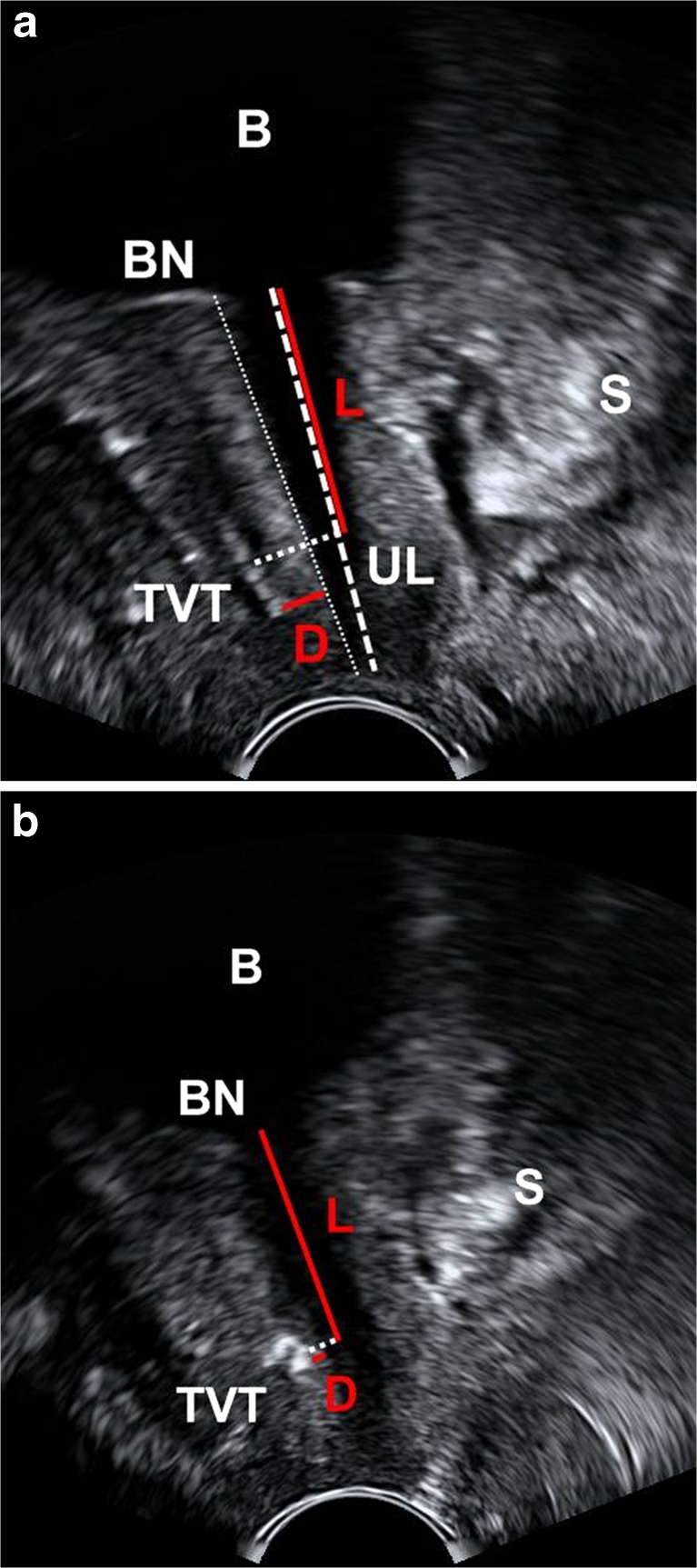
Ultrasound localization of the sling at rest for diagnosis of a dystopic sling or dysfunction. **a** Pelvic floor ultrasound image showing the optimal position of the tension-free vaginal tape (*TVT*) **b** Pelvic floor ultrasound image showing a TVT positioned too close to the urethra with excessive tension (sling curling). *B* bladder, *BN* bladder neck, *S* symphysis; *D* shortest distance between the sling and the longitudinal smooth muscle (LSM) complex; *UL* length of the entire urethra (bladder neck to urethral papilla) as measured on ultrasound; *L* proportion of the urethra (bladder neck to middle of the sling) used to determine TVT position along the urethra (%)

Using a standardized protocol [8], ultrasound examinations from 2003 until the end of 2009 were performed with a GE Voluson 730 (GE Healthcare, Chalfont St. Giles, UK) and with a GE E8 ultrasound device (vaginal scanner, 4.0–9.0 MHz, 160° beam angle) from the beginning of 2010. The ultrasound distance between the urethra and suburethral sling (Fig. 3) was measured using the following parameters described in our previous publications [6, 7, 9]: urethral length (UL) was measured on a median sagittal scan with 300-ml bladder filling from the bladder neck to the distal end of the hypoechogenic urethra (ending at the hyperechogenic urethral papilla). The position of the sling (L) along the urethra (mid-sling as the reference point) was determined and expressed as a percentage of the entire urethral length (L/UL). To characterize the proximity of the sling to the urethra, the shortest distance between the sling and the longitudinal smooth muscle (LSM) complex of the urethra, also called the sling–LSM distance, was measured. This was done by drawing a perpendicular line from the urethral lumen to the sling.

Following primary diagnostic assessment each patient received tailored, conservative management of their condition (elimination of infection, local estrogen, physical therapy or drug treatment). If this initial approach was insufficient, the option of suburethral sling incision was discussed with the patient. Patients who were successfully treated with conservative management were not included in this analysis. In order to be eligible for the study, patients had to have at least one of the six most commonly reported complications/indications for sling incision at our center: overactive bladder, voiding dysfunction, recurrent UTIs, dyspareunia, chronic pelvic pain and/or sling exposure [10].

**Fig. 4:**
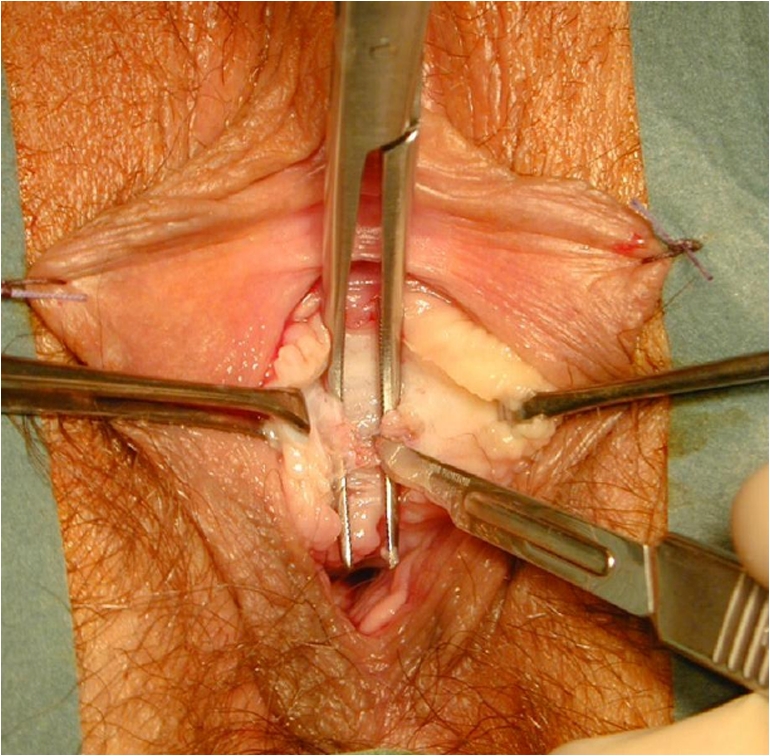
Intraoperative site of midurethral sling incision performed with the patient in the lithotomy position. It is important that the sling is severed completely since remaining fibers can cause additional complications (i.e., tissue penetration, obstruction). The sling is dissected suburethrally; the clamp is placed underneath the sling and slightly opened; the sling is then completely severed with scissors or a scalpel in between the arms of the clamp

The sling incision procedure (Fig. 4) was performed with the patient in the lithotomy position. In patients with sling exposure, the sling was localized visually and by palpation. Using information about sling position obtained from pelvic floor ultrasound, slings close to the urethra were then identified by intraurethrally palpating the sling edge with a Charrière 21 to 24 bougie à boule (Figs. 1, 2b). A tight sling with a stop cock mechanism was identified by withdrawing and rotating a No. 5 to 7 Hegar dilator through the urethra (snap mechanism at the site of the sling, Figs. 1, 2a). When performing the incision in analgosedation (Fig. 4), suburethral and periurethral local anesthesia were applied by using 20 ml of 1 % Xylonest (prilocaine hydrochloride) in conjunction with epinephrine. A short, suburethral, sagittal incision was made for blunt dissection of the sling with scissors pulled slightly forward and then completely severed with a slightly opened clamp placed underneath the sling (Fig. 4). After complete midline transection of the sling, the vaginal skin was closed with 3-0 vicryl single-buttress sutures. In cases of additional sling exposure, not only was the sling transected at the midline, but the exposed part was excised. All sling incision procedures were performed by two experienced urogynecologists. Patients left the operating room without a catheter and were treated in accordance with standard postoperative care. Initial attempts to void were made 2 h after the procedure. A bladder scan (BladderScan® BVI 6100, Verathon Medical Germany) was done after spontaneous voiding to measure the residual volume.

Postoperative follow-up visits scheduled at 2-, 6- and 12-week intervals were done to assess the patient’s condition regarding complications and SUI. However, for the purposes of this study the most pivotal visit for determining the health status of the patients was at 12 weeks. Follow-up examinations included a history of symptoms, clinical examination, measurement of residual urine volume, and pelvic floor ultrasound. SUI was assessed as previously described by Kociszewski et al. [6, 9] using a combination of objective and subjective criteria. Specifically, patients with a negative stress test, a negative 1-h pad test (less than 2 g) and a VAS score of 0–1 at 12 weeks’ follow-up evaluation were classified as being stress urinary continent. All other patients were classified as incontinent. Comprehensive urodynamic testing was performed only for patients undergoing incontinence surgery. Because of the recurrent nature of urinary tract infections, long-term urogynecological assessments (≥1 year) including urine cultures were conducted for patients with this complication.

The study concept was submitted to the local ethics committee, which waived the need for formal approval as only routine clinical data were required for analysis (reference 080605). Descriptive and inferential statistical analyses were done in SPSS version 19. Non-parametric (Pearson Chi-squared, Mann–Whitney *U*, and one sample binomial) and parametric (Student’s *t*) tests were conducted using a significance level of 0.05.

## Results

Data were obtained and assessed from 203 women who underwent sling incision at the tertiary urogynecology unit during this 8.5-year period. Of these 203 cases, 5 patients were excluded from the analysis since their sling incision procedure also included insertion of a new TVT. Therefore, a total of 198 women were included in this retrospective review. The majority of the women had the sling insertion procedure at another institution. However, 95 women underwent TVT™ or TVT-O™ (Ethicon, Somerville, MA, USA) insertion at our center, which accounts for approximately 5.6 % (1,696) of the total number of TVT insertion procedures performed during this time period. The types of slings most frequently used in all patients were TVT (70 %) and TVT-O (23 %). The remaining 7 % of the slings were distributed among Monarc™ (AMS, Minnetonka, MN, USA), Serasis® (Serag-Wiessner KG, Naila, Germany), Safyre (Promedon, Córdoba, Argentina), Sparc™ (AMS), TVT-Secur™, (Ethicon), and MiniArc (AMS).

The mean age of the women was 64 years (±12; range 36–89 years) and a median weight of 72.8 kg (range, 52–103) with a median body mass index (BMI) of 26.4 kg/m^2^ (range, 21–38). The median number of spontaneous deliveries was 2 (range, 0–6). The median interval between the sling insertion procedure and sling incision was 33 months (IQR 9–59 months). The mean duration of the surgical procedure was 19 min (±4; range 8–35 min). The range of blood loss during surgery was 10 to 90 ml with a median of 45 ml. There were no intra-interventional complications and post-interventional wound healing was uneventful. The only type of complication, a small hematoma, developed at the operative site in 13 patients and resolved spontaneously in all cases. The median length of hospitalization was 1.4 days (range 1–5).

All 198 women were seen at the pivotal 12-week follow-up visit at a mean of 12.2 weeks (±3.0) postoperatively. However, 10 (5 %) of the patients had incomplete data (ultrasound results) in their medical records. With regard to long-term data collection, 159 (80.3 %) women had additional urogynecological visits 52 weeks (±2) following the sling incision procedure. The last recorded visit was at a median of 82.5 weeks (IQR 53–166).

Overactive bladder (68 %), voiding dysfunction (61 %) and recurrent urinary tract infections (53 %) were the most frequently experienced complications reported by this study population. Cases of multiple complications were common among the women, with a median of two complications per patient (IQR 2–3 complications). Resolution rates from these six complications ranged from 60 to 100 %. More detailed descriptions of the findings for each of these complications are described below.

### Voiding dysfunction

**Fig. 5:**
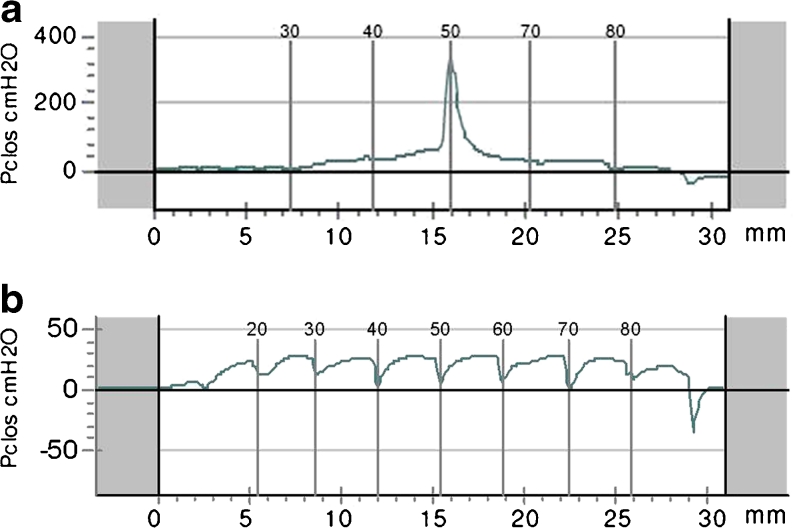
Urethral closure pressure profile (UCPP) during stress in patients with obstructive sling complications. **a** Before sling incision: a dramatic increase in urethral closure pressure during coughing is apparent at the site of the sling, resulting in continence during stress and obstructive complications (voiding dysfunction such as staccato micturition, urinary flow rate reduction, and elevated residual urine). **b** Postoperative profile after sling incision: sling incision eliminates the stop cock phenomenon, which results in normal micturition and residual urine, but stress urinary incontinence

Prior to sling incision, 120 women (61 %) had voiding dysfunction with an average residual urine volume of 210 ml. Sling incision relieved voiding dysfunction with normalization of residual urine (<100 ml) in 97 % (116) of these patients within 1–5 days (*p* < 0.0001). According to urodynamic examinations prior to sling incision, 80 % (96) of these 120 women showed a marked pressure increase at the site of the sling in the urethral closure pressure profile during coughing (positive pressure transmission ratios at the position of the sling attributed to urethral obstruction), consistent with a tonometric stop cock mechanism (Fig. 5) [11]. Probing with a Hegar dilator (Fig. 2a) revealed a snap phenomenon in 80.8 % (97) of the cases.

**Fig. 6:**
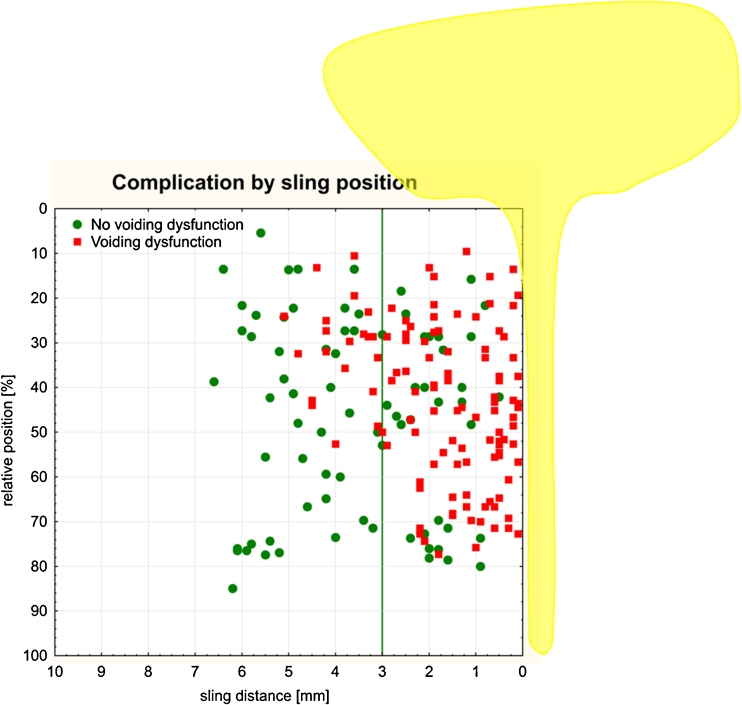
A scattergram summarizes the association of voiding dysfunction and sling location: sling distance to the LSM complex of the urethra and sling position along the urethra in patients (*n* = 188) with and without voiding dysfunction. Patients with a sling–LSM distance <3 mm were more likely to have voiding dysfunction (*p* < 0.0001)

A statistically significant difference in the sling–LSM distance of less than 3 mm was detected between patients with and without voiding dysfunction (*p* < 0.0001). For patients with voiding dysfunction, the median sling–LSM distance was 1.5 mm (IQR .60–2.5) and patients without voiding dysfunction had a median distance of 3.6 mm (IQR 2.05–5.1; Fig. 6). Of the patients with voiding dysfunction, the mean sling position was 43.13 % (±17.1) and those without voiding dysfunction had a mean position of 46.7 % (±21.5). No statistically significant difference was identified in the sling positions of these two groups (*p* = 0.208).

### Overactive bladder

The most frequently reported complication, overactive bladder, was experienced in 134 patients (68 %). OAB symptoms were resolved by sling incision in 80 of these 134 women (60 %; *p* < 0.0001). A sling–LSM distance of <3 mm was not associated with this complication (*p* = 0.24).

### Recurrent urinary tract infections

The third most commonly reported complication in over half (104; 53 %) of the women was recurrent urinary tract infections. Of these 104 patients, 77 (74 %) also had voiding dysfunction. At 12-week follow-up visits, no signs or symptoms of UTIs were detected in 86 % of the patients (89) following sling incision and concomitant antibiotic therapy (*p* < 0.0001). Of all the patients with urinary tract infections before sling incision, 87 (84 %) had a urogynecological visit 52 weeks (±2) following the sling incision procedure. Nineteen (22 %) of these women had urinary tract infections at these visits. When comparing women with and without recurrent urinary tract infections, statistically significant results were obtained in those women who had sling–LSM distances of <3 mm (*p* = 0.02). Moreover, no statistically significant differences in the sling position along the urethra were found in patients with recurrent urinary tract infections.

### Dyspareunia, chronic pelvic pain, and sling exposure

With regard to dyspareunia, 35 of the 198 women (18 %) experienced this symptom prior to sling incision. In 24 of these 35 patients (69 %), dyspareunia was due to sling exposure into the vagina. Of the 11 women with dyspareunia but no exposure, dyspareunia persisted in two cases. Overall, dyspareunia was resolved after sling incision in 33 women (94 %; *p* < 0.0001). Chronic pelvic pain in the area of the sling was reported in 33 of the 198 patients (17 %) before incision. Chronic pelvic pain was relieved after sling incision in 27 (81 %) of the 33 women (*p* < 0.0001). All 29 exposures were healed following the procedure, which included resection of sling ends in some patients (*p* < 0.0001). Statistically significant results were observed in patients with sling exposure. Patients with a sling–LSM distance <3 mm were less likely to have sling exposure (*p* = 0.004, OR 0.29 [95 % CI 0.122–0.693]).

### Recurrent SUI after sling incision

The stress urinary incontinence rate before sling incision was 29 % (58 out of 198). Statistically significant results were observed with a sling–LSM distance >5 mm (*p* < 0.0001). A total of 141 patients (72 %) were incontinent after the procedure. Of the 58 women who were incontinent prior to sling incision, 56 remained incontinent and 2 cases were lost to follow-up assessment of SUI status. In the sub-group that was continent prior to sling incision (140), 85 or 61 % developed recurrent SUI following the procedure.

## Discussion

Since suburethral sling insertion has emerged as the gold standard in the surgical treatment of female stress urinary incontinence [12], effective management strategies for the associated postoperative complications are becoming more important [13–15]. A distinction is needed, however, between the two main types of postoperative complications occurring after sling procedures—obstructive and non-obstructive. Obstructive complications (i.e., voiding dysfunction, overactive bladder) are more common and are encountered in approximately one in seven patients at some time after sling insertion. Non-obstructive complications (i.e., sling exposure and fistula formation) are less common, occurring in 1–2 % of women undergoing a sling procedure [2, 16, 17].

Given the higher incidence of obstructive complications, researchers have been investigating the causes of complications, such as the role of the sling position in the development of voiding dysfunction. Consistent with other earlier studies, findings from this retrospective analysis show a statistically significant difference in the proportion of women with voiding dysfunction who also had a sling–LSM distance <3 mm. A distance of the sling of more than 5 mm from the LSM complex was associated with SUI. Although the importance of the sling location has been disputed in the literature [7, 9, 18–21], mounting evidence suggests that too much tension placed on the sling as well as dystopic sling positioning (i.e., too proximal at the level of the bladder neck), should be avoided whenever possible.

Our findings indicate that most postoperative complications can be successfully managed with a sling incision, a minimally invasive procedure performed under analgosedation/local anesthesia [22–24]. Despite the apparent benefits of sling incision in treating postoperative complications, our results revealed a risk of recurrence of stress urinary incontinence following the procedure in as many as 61 % of the women, which is higher than previously published rates (i.e., 21 % by Molden et al.) [25]. The present study is distinct, however, in that it was conducted with a relatively large number of patients who had the same procedure (only midline incision) performed at one center and definitions of “cured patients” differed.

The shortcomings of our study were the limited data on a wider array of patient risk factors, and the inability to compare sling recipients who developed complications and were treated conservatively with those who had minimally invasive treatment for postoperative complications. Since over 50 % of the study patients (103) had sling insertion procedures at another institution, medical histories for many of these patients were incomplete. Consequently, data on preexisting conditions prior to sling insertion, such as OAB or voiding dysfunction, were not readily available. Yoost et al. attempted to identify factors that could predict an individual patient’s risk of recurrent SUI following sling incision. The authors found no associations from potential risk factors such as prior operations, amount of post-void urine or parity [26].

Additionally, recently published data from a small-scale, retrospective analysis of 15 patients who underwent a unilateral division of the sling, also known as a “J” cut, indicated that this type of division may carry a lower risk of SUI recurrence than the midline division [27]. The authors suggest that when the sling is divided unilaterally, support remains under the urethra and any excess tension is released. Unpublished data gathered by our institution on 41 women who underwent a unilateral division showed no significant differences in outcomes between those women with a midline or unilateral incision. These 41 women were not included in this analysis since the study was designed to assess a homogeneous group with a pure “midline incision” rather than compare two groups with different interventions. The lack of published data from a larger scale trial on the impact of the location of the incision, and the shortcoming of the above-mentioned study to only include patients with low bladder capacity limit generalizable conclusions.

In addition, researchers must rely primarily on retrospective rather than prospective data since the incidence of complications following sling insertion is relatively low. Even though outcome data used in this study were collected prospectively, with the secondary intent of assessing the role of pelvic floor ultrasound, our study design was largely retrospective. Despite these limitations, the prospects of a moderately high risk of recurrence of SUI compared with the benefits of the sling incision should be taken into account by both the physician and patient when sling incision (midline or unilateral) is the primary treatment option.

This risk, however, should not lead a patient to refuse a sling incision, when advisable, since recurrent incontinence can be effectively treated with individually tailored pessary treatment. Pessary treatment is a temporary measure used during an interval of approximately 3 months until a new sling procedure can be performed. Pessary treatment is often well-received by patients, particularly those distressed by the course of events following the initial procedure, and some women may even opt for long-term pessary treatment rather than another sling procedure. A recent study by Ala-Nissilä et al. demonstrated that the suburethral sling is also a valid option for women with recurrent SUI and outcomes are comparable to those of the primary sling procedures [28]. In another study with a 5-year follow-up period, Palva et al. found favorable results for repeat TVT procedures in recurrent SUI. In their study population of 26 women, 75 % were cured or had markedly improved [29].

In addition to this moderately high risk of SUI recurrence, a sling incision ought to be viewed as a *reactive* approach to managing postoperative complications. More *proactive* and timely strategies are needed to avoid undue distress in women with complications, especially late developing ones, which tend to be overlooked and/or their diagnosis delayed. As a consequence, the suffering of these women is prolonged as they undergo inadequate and expensive examinations before the problem can finally be identified and managed [30].

This raises the question of how to most effectively prevent and/or promptly detect postoperative complications. One approach is to focus more on practical diagnostic strategies, such as using widely available ultrasound technology pre- and postoperatively. Useful diagnostic information can also be obtained with simple tools such as a Hegar dilator or a bougie à boule (Figs. 1, 2). These tools are particularly helpful in detecting the more frequently occurring obstructive complications, especially those resulting from too tightly placed or dystopic slings. In addition, non-obstructive sling complications can also be readily detected by ultrasound. Given that slings placed too far from the posterior urethral wall and close to the vaginal skin may cause erosion or perforation of the vaginal skin resulting in infection, occult or visible abscesses, and soft tissue infiltration with fistula formation or ulceration, increasing the physician’s ability to place the sling within a desirable range ought to be a priority.

Although sling incisions should not be ruled out as a means of treating all complications following sling insertion, further research is needed to better understand the likelihood of SUI recurrence and the risk factors that play a role in the development of this discouraging and costly outcome. Moreover, research efforts should be aimed at exploring ways to decrease the likelihood of developing postoperative complications, primarily those associated with too tightly placed or dystopic slings resulting from the challenge faced by the physician to ensure a cure for stress incontinence while avoiding inadvertently causing overactive bladder and voiding problems. However, striving to fully eliminate the incidence of postoperative complications is unrealistic. Therefore, early detection and treatment of complications using practical, evidence-based technology such as pelvic floor ultrasound should be at the forefront of our efforts.
